# MRI characteristics of lobular carcinoma of the breast

**DOI:** 10.1186/bcr2993

**Published:** 2011-11-04

**Authors:** A Leaver, G Bristow, A Redman, J Potterton

**Affiliations:** 1 Gateshead Health NHS Foundation Trust, Gateshead, UK

## Introduction

Lobular carcinoma is frequently atypical on conventional imaging. MRI has increasingly been used to evaluate lobular carcinoma in recent years. Our experience has been that lobular MRI findings are also relatively difficult to interpret. In this study we aim to define lobular MRI characteristics to aid preoperative staging accuracy.

## Methods

This retrospective study included all lobular breast cancers which were subjected to preoperative MRI in our Trust from May 2010 to May 2011. Radiologists blinded to the original MRI reports used a questionnaire based on BIRADS to describe the findings. Final surgical histology was used as the gold standard for comparison. Descriptive statistics were performed on data obtained.

## Results

Full imaging and final surgical histology was available for 24 ladies with lobular carcinoma. In 83% (20/24) of cases the MRI tumour morphology was of a mass (19/20 irregular mass). Mass enhancement varied: heterogeneous 50% (10/20), homogeneous 40% (8/20). MRI 3D reconstruction gave the largest measurement most frequently (11/24), but the third post-contrast sequence most frequently (8/24) provided the most accurate tumour measurement. Enhancement curves were type 2 in 15/24 cases. In 21/24 cases the question of MRI multifocality correlated with final surgical histology. A total of 12.5% (3/24) patients had contralateral lesions identified by MRI.

## Conclusion

The most common lobular cancer morphology on MRI is an irregular mass with heterogeneous, type 2 enhancement. When compared with surgical histology, the third post-contrast (coronal) sequence gave the most accurate tumour measurement most frequently, with 3D reconstruction providing an overestimate in some cases.

